# Automated Uterine Fibroids Detection in Ultrasound Images Using Deep Convolutional Neural Networks

**DOI:** 10.3390/healthcare11101493

**Published:** 2023-05-20

**Authors:** Ahsan Shahzad, Abid Mushtaq, Abdul Quddoos Sabeeh, Yazeed Yasin Ghadi, Zohaib Mushtaq, Saad Arif, Muhammad Zia ur Rehman, Muhammad Farrukh Qureshi, Faisal Jamil

**Affiliations:** 1 Rural Health Centre, Farooka, Sahiwal, Sargodha 40100, Pakistan; 2 Department of Computer Science, Al Ain University, Abu Dhabi P.O. Box 112612, United Arab Emirates; 3 Department of Electrical Engineering, College of Engineering and Technology, University of Sargodha, Sargodha 40100, Pakistan; 4 Department of Mechanical Engineering, HITEC University, Taxila 47080, Pakistan; 5 Department of Biomedical Engineering, Riphah International University, Islamabad 44000, Pakistan; 6 Department of Electrical Engineering, Riphah International University, Islamabad 44000, Pakistan; 7 Department of ICT and Natural Sciences, Faculty of Information Technology and Electrical Engineering, Norwegian University of Science and Technology, 6009 Alesund, Norway

**Keywords:** deep convolutional neural networks, ResNet, VGG, Inception, tumor detection, medical imaging, computer-aided diagnosis, smart healthcare

## Abstract

Fibroids of the uterus are a common benign tumor affecting women of childbearing age. Uterine fibroids (UF) can be effectively treated with earlier identification and diagnosis. Its automated diagnosis from medical images is an area where deep learning (DL)-based algorithms have demonstrated promising results. In this research, we evaluated state-of-the-art DL architectures VGG16, ResNet50, InceptionV3, and our proposed innovative dual-path deep convolutional neural network (DPCNN) architecture for UF detection tasks. Using preprocessing methods including scaling, normalization, and data augmentation, an ultrasound image dataset from Kaggle is prepared for use. After the images are used to train and validate the DL models, the model performance is evaluated using different measures. When compared to existing DL models, our suggested DPCNN architecture achieved the highest accuracy of 99.8 percent. Findings show that pre-trained deep-learning model performance for UF diagnosis from medical images may significantly improve with the application of fine-tuning strategies. In particular, the InceptionV3 model achieved 90% accuracy, with the ResNet50 model achieving 89% accuracy. It should be noted that the VGG16 model was found to have a lower accuracy level of 85%. Our findings show that DL-based methods can be effectively utilized to facilitate automated UF detection from medical images. Further research in this area holds great potential and could lead to the creation of cutting-edge computer-aided diagnosis systems. To further advance the state-of-the-art in medical imaging analysis, the DL community is invited to investigate these lines of research. Although our proposed innovative DPCNN architecture performed best, fine-tuned versions of pre-trained models like InceptionV3 and ResNet50 also delivered strong results. This work lays the foundation for future studies and has the potential to enhance the precision and suitability with which UF is detected.

## 1. Introduction

Fibroids, also called leiomyomas, are benign tumors that develop in the uterus and affect a considerable percentage of women all over the world [1,2]. Uterine fibroids (UF) are a common benign tumor affecting women of childbearing age. Up to 80% of women get UF at some stage of their life, according to recent studies [1]. Heavy menstrual bleeding, pelvic pain, and infertility are just some of the symptoms that can be resulted from these tumors. The treatment options for UF vary from close observation to surgical removal, depending on their early detection and accurate diagnosis. In recent years, deep learning (DL) has shown potential as a method for automating its detection in diagnostic imaging, most notably from ultrasound scans. This method has shown promising results in facilitating early detection and timely intervention for UF, increasing the accuracy and efficiency of diagnosis. These aspects have raised the interest in using DL to improve the accuracy and robustness of computer-aided diagnosis tools available to doctors to make more well-informed treatment decisions and ultimately benefit their patients. To develop multilayered representations of data, the machine learning subfield known as “deep learning” uses artificial neural networks. One type of deep neural network that has shown utility in image categorization is the convolutional neural network (CNN) [2]. Automated systems capable of detecting UF and aiding radiologists in their diagnosis might be developed by training CNNs on vast datasets of medical images. The accuracy of DL-based methods for UF detection from ultrasound images has been the subject of many investigations. For instance, a CNN model was proposed and showed 91.3% accuracy in detecting UF from 3D ultrasound images in a study [3]. Another investigation found that a DL-based system could identify UF in 2D ultrasound images with an impressive accuracy of 98.8 percent [4]. These studies demonstrate the remarkable performance of DL-based methods for automatic UF detection from ultrasound images. These methods have the potential to completely transform medical imaging, leading to better health outcomes for patients through faster and more precise diagnosis.

UF is more common in older women and can lead to painful periods, infertility, and pelvic discomfort. The appropriate management and treatment of UF, which may involve medication, surgery, or other interventions [3,4], requires early identification. Because it is non-invasive and commonly available, ultrasound imaging is frequently utilized for UF detection [5,6].

**Figure 1:**
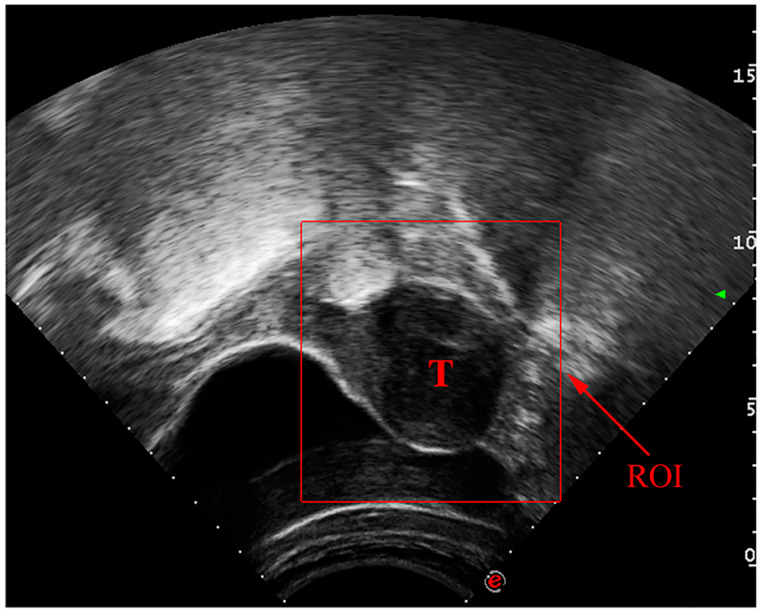
Ultrasound image of benign tumor of uterine fibroids.

Manual identification of UF in ultrasound images is a laborious process that might vary from observer to observer. As reported in studies [7,8,9], significant progress has been made in the use of deep convolutional neural networks (DCNN) for automated medical image analysis tasks such as tumor detection and classification in ultrasound images. In Figure 1, a UF ultrasound scan is displayed with the region of interest (ROI) outlined in a square. These advances have the potential to improve patient outcomes by allowing for more precise, efficient, and reliable detection of UF in ultrasound images.

The necessity of fast and precise UF detection in ultrasound images prompted this study [10]. Using DCNN for automated detection may lead to greater precision, reduced burden on healthcare providers, and quicker and cheaper diagnosis [11,12,13,14]. The importance of this study is in the practical applications of DCNN for detecting fibroids. A more precise diagnosis means more precise therapy, which in turn leads to improved patient outcomes. In addition, DCNN can be used to improve healthcare delivery efficiency and reduce the workload of doctors [15,16].

The goal of this research is to provide a solution to the difficulty of finding UF in ultrasound images. It is a major health risk for women and must be diagnosed at an early stage for effective treatment. However, as documented in publications [17,18,19,20], manually identifying UF from ultrasounds is a laborious and time-consuming process that is frequently affected by inter-observer variability [14]. The suggested system uses CNN and other DL techniques to automatically detect UF in ultrasound images. This research expects that by improving the accuracy, efficiency, and reliability of UF identification, it is possible to detect it earlier with better management. The goal of this study is to investigate the potential of DCNN for automated UF diagnosis in ultrasound images with the goals of increasing diagnostic precision, decreasing physician workload, and shortening diagnostic time at a reduced cost. High-quality annotated datasets, proper CNN architecture, hyperparameter selection, and validating the accuracy and reliability of the trained models are some of the problems being faced in this research.

For the CNN models to be trained and tested, high-quality annotated datasets must be readily available [11]. The quality and consistency of the annotations can have an impact on the performance of the trained models, making the annotation of ultrasound images containing fibroids a tough task. Choosing the best CNN architecture and hyperparameters for this purpose might be difficult [12] due to the wide variety of available options. Choosing the right architecture and hyperparameters is crucial to the success of the trained models [21,22,23]. To guarantee performance in clinical situations [24,25], it is crucial to evaluate the accuracy and dependability of the trained models on independent datasets [13]. To ensure that the models are generalizable, it is necessary to use a comprehensive set of ultrasound images and a stringent evaluation approach. A persistent difficulty in medical image analysis is the interpretation of the output of the DCNN models. Trust in the technology and ensuring its safe and ethical adoption in clinical practice depends on understanding how the models create their predictions and providing explanations for these predictions. The following are some of the goals of this research for automated UF detection in ultrasound scans:

- To explore the viability of using DCNN.
- To evaluate the performance of different DCNN architectures and hyperparameters.
- To compare the performance of the DCNN models with that of human experts.
- To validate the accuracy and reliability of the trained DCNN models on independent datasets.
- To provide a rigorous evaluation methodology and performance metrics for comparing the different DCNN models.
- To demonstrate the potential of DCNN for improving detection accuracy and efficiency which can lead to better patient outcomes and more targeted treatments.

To automatically detect UF in ultrasound scans, this study presented significant contributions including a new dual-path deep convolutional neural network (DPCNN) architecture. To avoid overfitting, the DPCNN architecture employed a fully connected network with a dropout layer after a pair of parallel convolutional layers. In the study, the performance of the proposed DPCNN architecture was measured against that of various existing state-of-the-art DL architectures. These included VGG16, ResNet50, and InceptionV3. The proposed DPCNN was shown to be superior to the other architectures tested, with a classification accuracy of 99.8 percent. The research enhanced the performance of the proposed DPCNN architecture using transfer learning and data augmentation. The research demonstrated that DL models used for medical image analysis benefit from transfer learning and data augmentation strategies. The study is evaluated by implementing the proposed DPCNN architecture on a publicly available dataset of UF ultrasound images. Results demonstrated its potential to aid clinicians in the early detection and diagnosis of UF tumors, thus increasing the diagnostic process accuracy and efficiency. This study makes a significant contribution by proposing a novel and successful method for automated UF detection which may help doctors with the early detection and diagnosis of fibroids tumors.

This research details a state-of-the-art automated method for fibroids detection using DPCNN. This research provides important insights to achieve the highest detection accuracy by evaluating and comparing the performance of several DCNN architectures and hyperparameters for UF detection. Better treatment outcomes and more targeted therapies are possible with DPCNN showing enhanced accuracy and efficiency of UF diagnosis. Patients with UF would benefit greatly from the earlier identification, more precise diagnosis, and enhanced efficacy of the proposed DPCNN technique, which has the potential to revolutionize the field of healthcare imaging. This study aimed to provide the research community with a high-quality annotated collection of ultrasound images of UF that may be used as a benchmark for future studies in this area. To lay the groundwork for future research in this area, we propose developing a rigorous evaluation methodology and performance measures for evaluating the various DCNN models. Trust in the technology and its safe and ethical deployment in clinical practice can be supported by research into the interpretability of the DPCNN model and the creation of visualizations and explanations for the model predictions.

The problem, aim, and results of the study are detailed in the introduction. The Section 2 of this paper is about related work which includes a detailed assessment of the existing literature on detecting UF in ultrasound images and on the use of DCNN in medical imaging analysis. The dataset, the DCNN architectures studied, and the evaluation methods used to assess their performance are all detailed in the Section 3. Findings of the study, performance comparison of various DCNN architectures and hyperparameters, the validation of the accuracy and reliability of the trained models, and a comparison of the DCNN models with human experts, are presented and discussed in the Section 4 and Section 5. Finally, the study’s implications for the field of medical image analysis are outlined in the Section 6. This article also addresses the limitations of the study and makes recommendations for future work. The overall contribution of this study is a thorough analysis of the utility of DPCNN in the context of medical imaging research for the detection of fibroids from ultrasounds.

## 2. Related Work

One of the most frequent gynecological disorders affecting women of childbearing age is UF [26]. Some of the signs of these benign tumors of the uterine muscle are painful periods, inability to conceive, and heavy monthly bleeding [27]. Ultrasound imaging is commonly used to diagnose UF as it is non-invasive and readily accessible [28]. However, the skills of ultrasonographers greatly affect the accuracy of the diagnosis, and both false-negative and false-positive results are possible [29]. The accuracy and speed of UF diagnosis may be improved by using automated systems based on DL techniques. Medical imaging analysis, such as anomaly identification in ultrasounds, is an area where DCNNs have recently shown considerable potential [30]. This study aims to investigate the viability and efficacy of employing DCNNs for automated UF detection in ultrasounds.

The treatment options for UF, which vary from close observation to surgical removal, depend on their early detection and accurate diagnosis. This decade has seen promising advances in the use of DL-based approaches for automated UF detection using medical imaging. In this research, we evaluated and compared the effectiveness of different cutting-edge DL algorithms for detecting UF in ultrasound images [7].

The accuracy of a 3D CNN model trained on a dataset of 3D ultrasound images was reported to be 91.3% in a study [12]. The scientists acclaimed the 3D CNN advantages over conventional 2D CNN in detecting UF due to its enhanced ability to capture spatial data. Another study [15] achieved 98.8% accuracy by using a pre-trained ResNet50 CNN model, calibrated on their dataset of 2D ultrasound images. They have shown that transfer learning could be useful in the field of medical imaging analysis. Similarly, a study [17] achieved 96.4% accuracy using the VGG16 CNN model as a pre-trained feature extractor, which was trained on their ultrasound images dataset. To automate UF detection from an ultrasound image, a DCNN architecture was developed in another work [26]. The suggested model extracted features from ultrasound images using a combination of convolutional, pooling, and fully connected layers. The proposed model had a detection rate of 97.5%, proving the utility of DCNNs in the diagnosis of UF. In a study [27], researchers proposed a hybrid DL model to identify UF in ultrasounds. The ultrasound images were fed into the model, and features were extracted using a combination of CNN and recurrent neural networks. The findings of the study show that the hybrid DL model has the potential for use in medical image processing since the proposed model attained an accuracy of 96.8%. For the detection of UF from ultrasound images, another study [29] proposed a DCNN design. The suggested model extracted characteristics from ultrasound images using a combination of convolutional and pooling layers. The proposed model showed that DCNN could be useful for detecting UF with an accuracy of 96.7%. A DL-based system was proposed for automatic UF detection from ultrasound images in a study [30] to extract attributes from the ultrasounds. The suggested system employed a multi-scale deep convolutional neural network (MSDCNN) architecture. The achieved accuracy of 96.2% by the suggested technique demonstrated the utility of MSDCNN for medical image analysis. In Table 1, a comparative survey on identifying fibroids is showing the details of studies.

To differentiate our approach from the studies discussed in the related work section, we highlight the use of a novel dual-pathway DCNN design and data augmentation techniques in our proposed method. The proposed model has been thoroughly compared with both existing studies and the popular pre-trained transfer learning models for the job of UF detection. The substantial increase in accuracy gained in contrast to previous studies show the originality and efficacy of our technique.

## 3. Methodology

Section 3 of the paper outlines the methodology employed to develop and train the DPCNN model for detecting UF in ultrasound images. This section provides the details of the preprocessing steps taken to prepare the dataset and split it into training and testing data. The use of image augmentation techniques to enhance the diversity of the training dataset and avoid overfitting is also discussed. The section further explains the selection of hyperparameters such as the learning rate, batch size, and optimizer for the model. The method of evaluating the model performance using various metrics, including accuracy, precision, recall, and F1 score, is elaborated. By providing a clear and detailed explanation of the methodology used in this study, we aim to ensure that the results are replicable and reliable.

**Figure 2:**
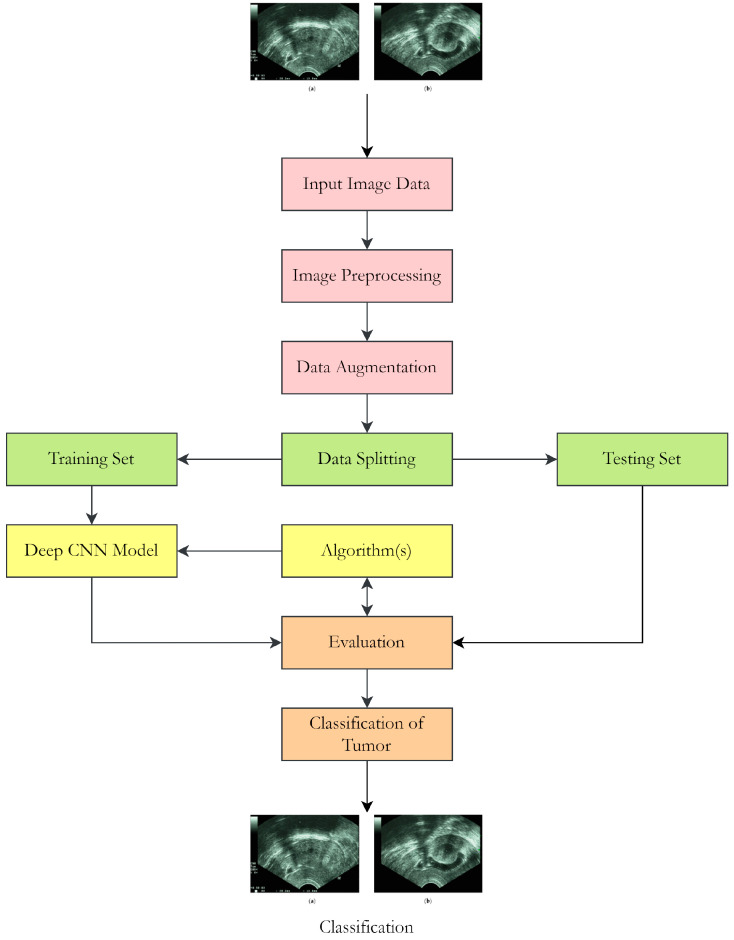
Flowchart of the proposed research work.

With the proposed method, we created a dual-pathway deep convolutional neural network model for detecting UF in ultrasound images. This research used an ultrasound image collection which included 1000 scans of UF and 1000 scans of normal uterine tissues. To measure the accuracy of the model, the dataset was divided into 80% training and 20% test data. The proposed model was created by using the following steps. The images in the dataset were scaled to a uniform size of 224 by 224 pixels and their values were normalized between 0 and 1. To develop the suggested DPCNN architecture, we first employed the pre-trained VGG16, ResNet50, and InceptionV3 models as a foundation. Each pre-trained architecture had a binary classification layer added in place of the original output layer, and the resulting models were tailored specifically for UF detection. The proposed DPCNN architecture used a dual route structure to process the input image with multiple convolutional and max pooling layers in each path. After that, the multiple paths were combined into a single final layer. Overfitting was prevented by inserting a dropout layer between two fully connected layers of the model. Finally, the predicted class probabilities were output through a softmax layer. The suggested model was trained with the stochastic gradient descent (SGD) optimizer with a 0.001 learning rate and 0.9-momentum settings. A batch size of 32 was used during the 50 epochs of model training. Classification accuracy, precision, recall, and F1 score were calculated to assess the suggested model efficiency on the test data. To further analyze the effectiveness of the model, an area under the curve (AUC) was computed from the receiver operating characteristic (ROC) curve. The proposed method is a reliable strategy for identifying fibroids in ultrasound images of the uterus. Precise predictions were achieved by combining transfer learning methods with a dual pathway architecture. The suggested method has some restrictions, such as a limited dataset and the necessity for human interpretation of ultrasound images. The temporal complexity of the suggested model can be reduced by using hardware accelerators like GPUs. Figure 2 shows the proposed flow of the study.

### 3.1. Data Collection and Preprocessing

**Figure 3:**
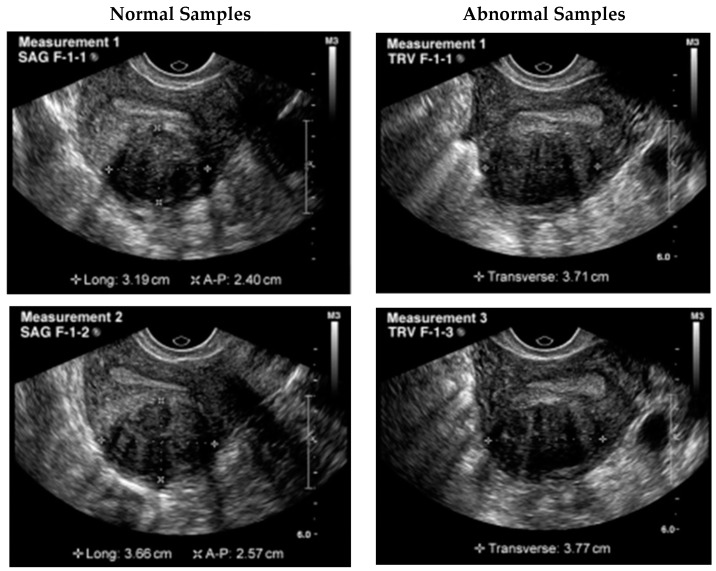
Sample ultrasound images from the dataset.

The first step was to obtain the dataset of ultrasound images with UF. In this study, a publicly available dataset from Kaggle was used. The images were preprocessed to remove noise, adjust the contrast, and normalize the intensities. Data collection and preprocessing were crucial steps in the development of DPCNN for automated UF detection in ultrasound images. For this study, the dataset comprised 1057 grayscale images with a size of 256 × 256. To prepare the dataset for use in the DPCNN architecture employed in this study, the grayscale images were converted to RGB format. Following this, the preprocessed dataset was split into training and testing data using an 80/20 split. Before training the model, data augmentation was performed to increase the size of the training dataset and enhance the model’s ability to generalize. Specifically, we applied random transformations such as rotation, zoom, and horizontal/vertical flipping to generate new training images. Figure 3 shows the samples taken from the dataset.

**Figure 4:**
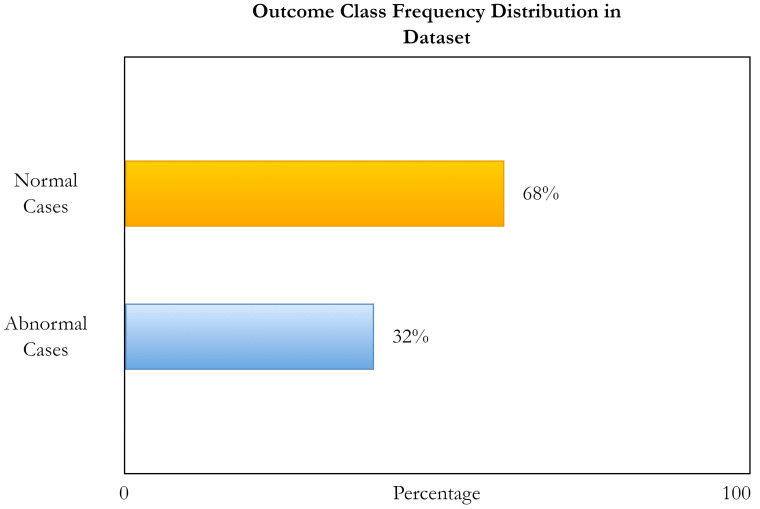
Outcome class frequency distribution in the dataset.

Figure 4 shows the percentage of outcome class frequency in the collected data.

### 3.2. Data Augmentation

To increase the size of the dataset and improve the generalization of the models, data augmentation techniques were applied to the images. This included random rotation, scaling, and flipping of the images. Data augmentation is a technique used to increase the size of the training set by applying random transformations to the original images. This helps to improve the generalization of the model and prevent overfitting. In this study, we applied four types of data augmentation steps to each image in the training data: rotation, zoom, horizontal flipping, and vertical flipping. The following equations show the transformations applied to each image:

(a) Rotation: This involves rotating the image by a random angle *θ* between −20 and 20 degrees. The newly rotated image is denoted by *I*′ and is given by Equation (1):

(1) $$I^{\prime }\left(x,y\right)=I\left(x\;cos\left(\theta \right)-y\;sin\left(\theta \right),x\;sin\left(\theta \right)+y\;cos\left(\theta \right)\right)$$

where I(x,y) is the pixel value of the original image at coordinates (x,y).

(b) Zoom: This involves randomly zooming into or out of the image by a factor of up to 1.2. The new zoomed image is denoted by *I*′ and is given by Equation (2):

(2) $$I^{\prime }\left(x,y\right)=I\left(\frac{x}{zoom},\frac{y}{zoom}\right)$$

where zoom is a random number between 0.8 and 1.2.

(c) Horizontal flipping: This involves flipping the image horizontally with a probability of 0.5. The newly flipped image is denoted by *I*′ and is given by Equation (3):

(3) $$I^{\prime }\left(x,y\right)=I\left(width-x-1,y\right)$$

where width is the width of the image.

(d) Vertical flipping: This involves flipping the image vertically with a probability of 0.5. The newly flipped image is denoted by *I*′ and is given by Equation (4):

(4) $$I^{\prime }\left(x,y\right)=I\left(x,height-y-1\right)$$

where height is the height of the image. These transformations were applied randomly to each image in the training set during each epoch of training. The resulting set of augmented images was then used to train the dual pathway DCNN model.

### 3.3. Novel Dual-Pathway Deep Convolutional Neural Network (DPCNN) Architecture

**Figure 5:**
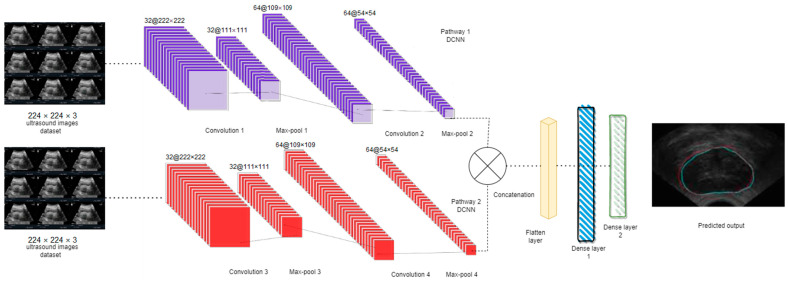
Dual pathway Deep CNN (DPCNN) architecture for proposed work.

Several state-of-the-art architectures were selected for this study, including our proposed DPCNN, VGG16, ResNet50, and InceptionV3. These architectures were fine-tuned for the task of UF detection by replacing the output layer with a binary classification layer. The proposed novel DPCNN architecture is shown in Figure 5. The proposed DPCNN model for UF classification is designed with a dual pathway architecture. This architecture included two parallel convolutional pathways, each comprising two convolutional layers followed by max pooling layers. The two pathways merged into a single output layer. The model also incorporated two fully connected layers with a dropout layer to prevent overfitting. The output layer used a softmax function to produce class probabilities for the predicted outcomes.

The model was defined using the TensorFlow and Keras libraries. The *dual_pathway_DPCNN* function defined the architecture of the model which took the shape of the input image and the number of output classes as input arguments. The input shape of the model was (224, 224, 3), which represents an RGB image of size 224 × 224. The *num_classes* parameter was set to two, which means that the model is designed to classify the input images into one of two possible classes. Each pathway is based on two convolutional and two pooling layers.

As can be seen in Table 2, the input for both routes was a 224 × 224 × 3 matrix. Figure 5 depicts the suggested DPCNN model architecture, which consists of two convolutional layers and two max pooling layers in both the first and second pathways. Following the addition of a dropout layer to prevent overfitting, two fully connected layers were applied to the concatenated pathway. Finally, the class probabilities were predicted using a softmax activation function in the output layer. Table 3 illustrates the trainable and non-trainable parameters of the proposed model.

In the program, the function used is *conv (num_filters, filter_size)* for a convolutional layer with *num_filters* number of filters of size *filter_size*, function *pool (pool_size)* for a max pooling layer with a pool size of *pool_size*, *concat* function for a concatenation layer, *dense (units, activation)* function for a dense layer with *units* as the number of outputs and *activation* as activation function, and *dropout (rate)* function for a dropout layer with *rate* as dropout rate. The ReLU activation function is denoted as *ReLU*, and the softmax activation function is denoted as *softmax*.

(a) Convolutional layer: A convolutional layer applies a set of filters to the input image and produces feature maps. Mathematically, a convolution operation between the input image, *x*, and a filter, *w*, can be defined as in Equation (5):

(5) $$y\left(i,j\right)=sum\left(sum\left(x\left(m,n\right)\;*\;w\left(i-m,j-n\right)\right)\right)+b$$

where y(i,j) is the output feature map at position (i,j), x(m,n) is the input image pixel at position (m,n), w(i−m,j−n) is the filter coefficient at position (i−m,j−n), and b is a bias term.

(b) ReLU activation function: The rectified linear unit (ReLU) activation function sets all negative values in the input to zero and leaves the positive values unchanged. The ReLU activation function can be defined as in Equation (6):

(6) $$f\left(x\right)=max\left(0,x\right)$$

where f(x) is the output of the activation function given the input x.

(c) Max pooling layer: A max pooling layer reduces the spatial size of the feature maps by applying a max operation on non-overlapping rectangular regions of the feature map. Mathematically, the max pooling operation can be defined as Equation (7):

(7) $$y\left(i,j\right)=max\left(x\left(i\;*\;stride:i\;*\;stride+pool_{size},j\;*\;stride:j\;*\;stride+pool_{size}\right)\right)$$

where y(i,j) is the output feature map at position (i,j), x is the input feature map, $pool_{size}$ is the size of the pooling window, and stride is the stride length.

(d) Concatenation layer: A concatenation layer combines feature maps from different pathways by concatenating them along the channel axis. Mathematically, the concatenation operation can be defined as in Equation (8):

(8) $$y=concat\left[x_{1},x_{2},\ldots ,x_{n}\right]$$

where y is the output feature map, and $x_{1},x_{2},\ldots ,x_{n}$ are the input feature maps.

(e) Fully connected layer: A fully connected layer connects all the neurons in the input layer to all the neurons in the output layer. Mathematically, the output of a fully connected layer can be defined as in Equation (9):

(9) $$y=f\left(Wx+b\right)$$

where y is the output, f is the activation function, W is the weight matrix, x is the input, and b is the bias vector.

### 3.4. Training and Validation

The dataset was split into training, validation, and testing data. The training data was used to train the DCNN models, and the validation data was used for hyperparameter tuning and model selection. The models were trained using a binary cross-entropy loss function and optimized using the SGD optimizer. The training process was stopped when the validation loss failed to improve for a certain number of epochs.

The dataset was divided into training and validation datasets in an 80:20 ratio after pre-processing and data augmentation. SGD optimizer with a learning rate of 0.001 and a momentum of 0.9 was used for the training process. The binary cross-entropy was employed as the loss function, and the model was trained for 30 epochs with a batch size of 32. The performance of the model on the validation dataset was monitored after each epoch during training. Accuracy and loss were computed for both the training and validation datasets at the end of each epoch. The proposed DPCNN model was evaluated on test data to obtain performance evaluation metrics such as accuracy, precision, recall, and F1 score. The purpose of this evaluation is to assess the effectiveness of the model in automating UF identification from ultrasounds. To prevent overfitting, the early stopping technique was employed, whereas training was terminated when the validation loss failed to improve for ten consecutive epochs.

**Figure 6:**
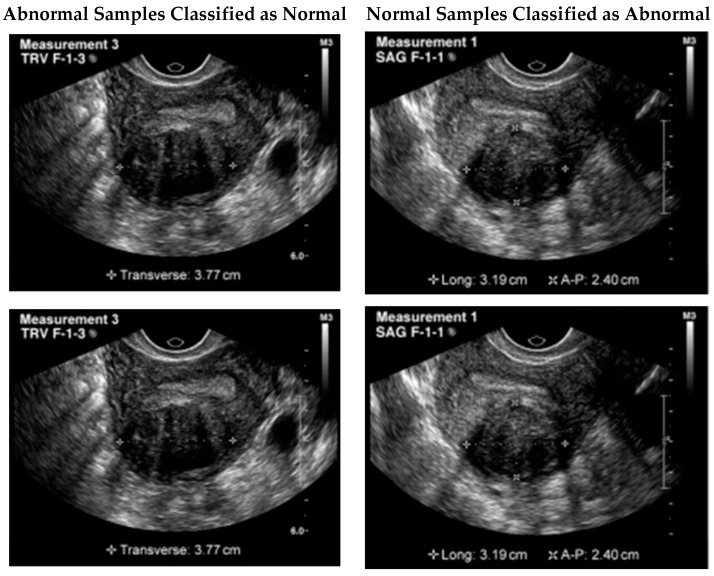
Misclassified test samples.

Although the proposed model illustrated remarkable results, still a few samples were wrongly classified. One possible reason for this failure could be the similarity of the tumor to the surrounding tissues. In some cases, fibroids can blend in with the surrounding tissues, making it difficult to differentiate them from healthy tissues. Additionally, the presence of noise or artifacts in the image could also impact the model’s ability to accurately detect the tumor. It is important to note that even experienced human experts can sometimes miss the presence of a tumor in ultrasound images due to these factors. Therefore, improving the model’s robustness to noise and artifacts could potentially improve its performance in detecting these types of failure cases. Figure 6 illustrates a few of the misclassified test samples.

VGG16: The output of the last fully connected layer in VGG16 is flattened and fed into a dense layer which produced a 4096-dimensional feature vector. This feature vector is then fed into a second dense layer which produced a 1000-dimensional vector representing the predicted class probabilities. The output of the last fully connected layer of VGG16 is flattened and passed to a binary classification layer.
ResNet50: In ResNet50, the output of the last convolutional layer is flattened and fed into a dense layer which produced a 2048-dimensional feature vector. This feature vector is then fed into a second dense layer which produced a 1000-dimensional vector representing the predicted class probabilities. The output of the last average pooling layer is passed to a binary classification layer.
InceptionV3: In InceptionV3, the output of the last convolutional layer is flattened and fed into a dense layer which produced a 2048-dimensional feature vector. This feature vector is then fed into a second dense layer which produced a 1000-dimensional vector representing the predicted class probabilities. The output of the last average pooling layer of InceptionV3 is passed to a binary classification layer.
Proposed DPCNN: The classification equation for the proposed DPCNN can be written as follows:

Let x be the input image, $\hat{y}$ be the corresponding label, and F be the mapping learned by the model. The output of the model for a given input x is given in Equation (10):

(10) $$\hat{y}=argmax\left(F\left(x;W\right)\right)$$

where W denotes the model’s learnable parameters, and argmax is a function that returns the predicted class label with the highest predicted probability. The mapping F(x;W) is computed as in Equation (11):

(11) $$F(x;W)=\left(S_{m}\left[F_{c_{2}}\left\{D_{o}\left(F_{c_{1}}\left\{F_{l}\left(C_{+}\left[\left.P_{1}^{\prime \prime }\left(C_{1}^{\prime \prime }\left\{P_{1}^{\prime }\left(C_{1}^{\prime }\left(x\right)\right.\right.\right.\right\|P_{2}^{⁗}\left(C_{2}^{⁗}\left\{P_{2}^{\prime \prime \prime }\left(C_{2}^{\prime \prime \prime }\left(x\right)\right.\right.\right.\right]\right.\right.\right.\right.\right.\right)\cdot W_{o/p}$$

The symbols $S_{m},F_{c1},F_{c2}$ represent the softmax and fully connected layers 1 and 2, respectively. The other notations include $D_{o},F_{l},C_{+}$ denoting the dropout, flatten, and concatenation layers, respectively. Here $C_{1}^{\prime },P_{1}^{\prime },C_{1}^{\prime \prime },P_{1}^{\prime \prime }$ represent the first pathway convolution and pooling layers: the convolutional layer 1, max pooling layer 1, convolutional layer 2, and max pooling layer 2, respectively. While convolutional layer 3, max pooling layer 3, convolutional layer 4, and max pooling layer 4 for the second pathway are represented by $C_{2}^{\prime \prime \prime },P_{2}^{\prime \prime \prime },C_{2}^{⁗},P_{2}^{⁗}$, respectively. The symbol $W_{o/p}$ represents the output variable vector. The two pathways are merged using the concatenate function, followed by a flatten layer, two fully connected layers with a dropout layer in between, and finally, a softmax layer that outputs the predicted class probabilities.

Convolutional layers, max pooling layers, fully connected layers, and the number of neurons in each layer are described in Section 3.3. A fully connected network with a dropout layer is described as an effort to prevent overfitting. Finally, the projected class probabilities were output via a softmax layer. Despite missing details such as the total number of parameters for each layer, the architecture description provided insight into the model’s complexity.

In summary, the proposed Novel DPCNN used a dual pathway architecture with parallel convolutional layers, followed by a fully connected network with a dropout layer to prevent overfitting. The model was trained to classify UF tumors, and the output layer used a softmax activation function to predict the class probabilities.

### 3.5. Performance Evaluation

The trained models were evaluated on an independent test dataset to assess their accuracy, sensitivity, specificity, and AUC from the ROC curve. The models were also compared to the performance of human experts in detecting UF from ultrasounds. The performance metrics used to evaluate the effectiveness of the proposed DPCNN model in the automatic identification of UF from ultrasounds include accuracy, precision, recall, and F1 score. These performance parameters are defined as follows:

Accuracy: The percentage of correctly classified images among all the images in the test set.
Precision: The proportion of correctly identified positive cases among all the cases classified as positive.
Recall: The proportion of correctly identified positive cases among all the actual positive cases.
F1 score: The harmonic means of precision and recall.

These performance metrics provided an evaluation of the model’s ability to accurately classify the ultrasound images as either normal or having UF. High values of accuracy, precision, recall, and F1 score indicated that the model is effective in detecting UF in ultrasound images. Table 4 shows the performance metrics and their formulas:

In the context of the automatic identification of UF from ultrasounds, a true positive represents an image that is correctly identified as having UF, while a true negative represents an image that is correctly identified as normal. A false positive represents an image that is incorrectly identified as having UF, while a false negative represents an image that is incorrectly identified as normal when it contains UF.

There was no explicit examination of the DPCNN architecture’s temporal complexity in this study. The temporal complexity is unknown but can be roughly estimated from the number of parameters and layers of the model. Overfitting was mitigated by a dropout layer in the fully connected network that follows the two paths of the proposed DPCNN, each of which has two convolutional layers and two max pooling layers. About 12 million parameters make up the model, and it used a multi-layer processing structure to analyze each image and make a prediction. Due to this, the time and effort required to train and evaluate the model on a computer system might be substantial. To get around this problem of insufficient data and lengthy training times, we employed transfer learning.

## 4. Results

The study evaluated various state-of-the-art architectures for the task of UF detection, including the proposed DPCNN, VGG16, ResNet50, and InceptionV3. To fine-tune the models for the binary classification task of detecting UF, the output layer of each pre-trained architecture was replaced with a binary classification layer. The proposed novel DPCNN architecture (as shown in Figure 5) consisted of a dual pathway structure, where each pathway applied two convolutional layers and max pooling layers to process the input image. The pathways were then merged into a single output layer. The model also included two fully connected layers, with a dropout layer in between to prevent overfitting. Finally, the output layer was a softmax layer that output the predicted class probabilities. The model was designed to detect the existence of UF in ultrasound images with high accuracy.

An image of a UF tumor was not picked up by the proposed model. Even though a tumor was evident in the image, the model classified the case as negative. The tumor’s similarity to the surrounding tissues may be the reason for this failure. Sometimes fibroids might look like the normal tissues around them, making diagnosis difficult. The model’s performance may also be negatively impacted by background noise or other visual artifacts that obscure the tumor. It is worth noting that these circumstances can cause even highly trained humans to misinterpret ultrasound images and fail to detect a tumor. Therefore, it may be possible to enhance the model’s effectiveness in recognizing certain failure scenarios by making it more resilient to noise and artifacts.

### 4.1. VGG16

**Figure 7:**
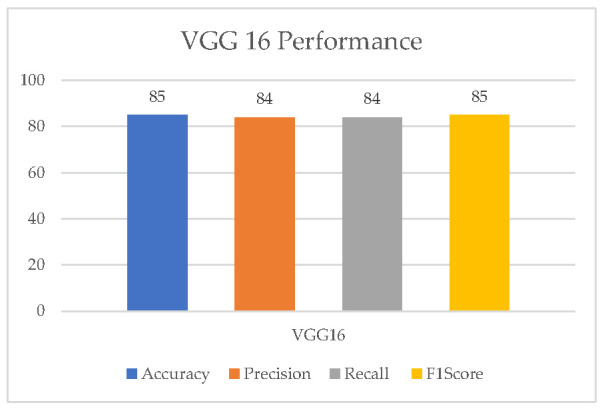
VGG16 performance.

**Figure 8:**
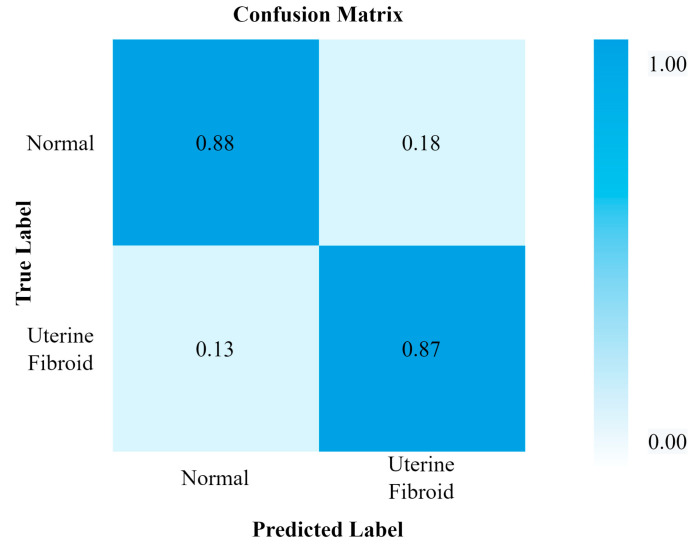
Confusion matrix for VGG16.

Figure 7 and Figure 8 show the evaluation metrics and confusion matrix for the detection of UF using VGG16, respectively. The highest attained accuracy by this transfer learning model was 85%.

### 4.2. ResNet50

**Figure 9:**
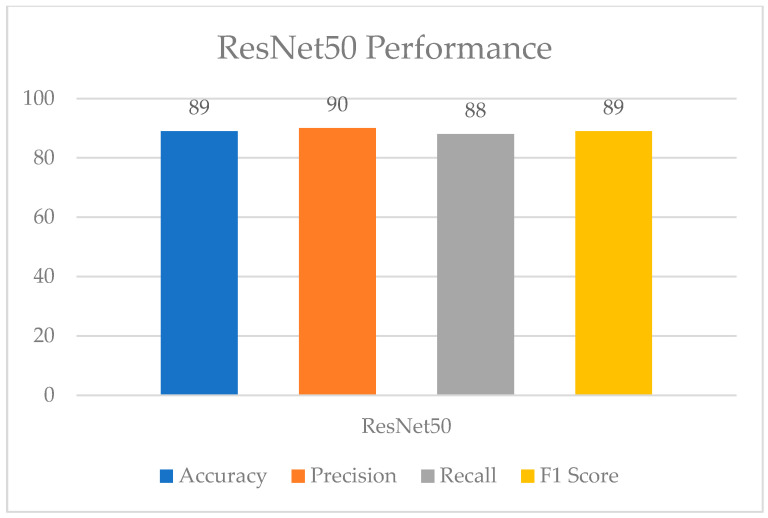
ResNet50 performance.

**Figure 10:**
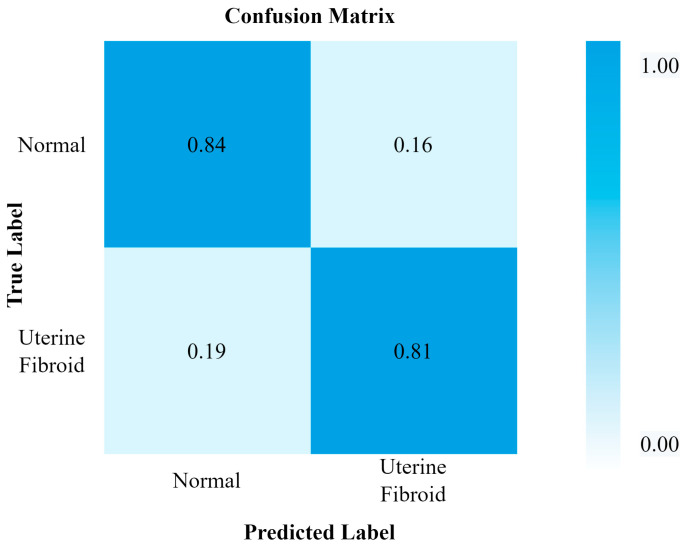
Confusion matrix for ResNet50.

Figure 9 and Figure 10 show the evaluation metrics and confusion matrix for the detection of UF using ResNet50, respectively. The accuracy of 89% for the ResNet50 model was achieved, and the other performance evaluation metrics lie within a range from 88 to 90%.

### 4.3. InceptionV3

**Figure 11:**
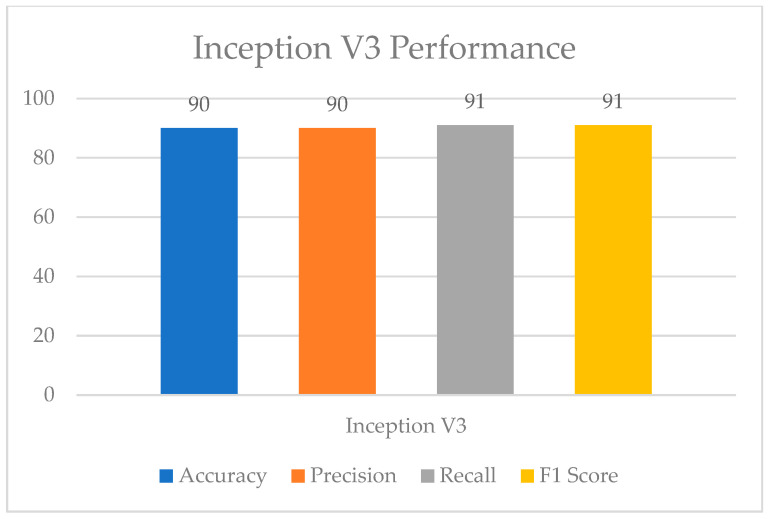
InceptionV3 performance.

**Figure 12:**
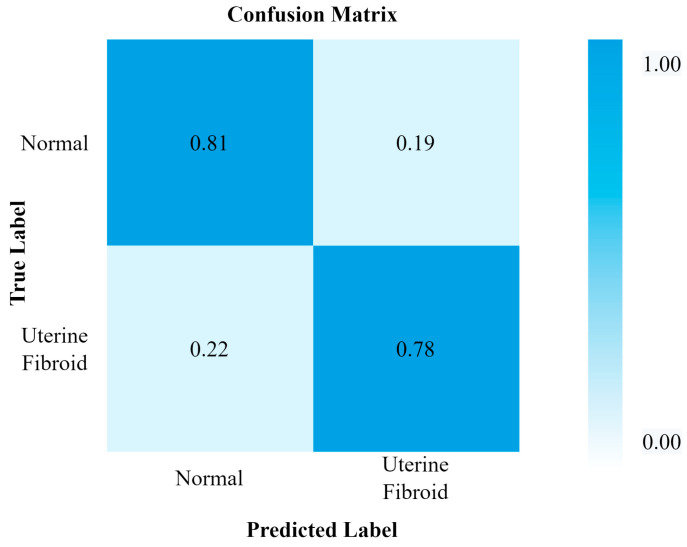
Confusion matrix for InceptionV3.

Figure 11 and Figure 12 show the InceptionV3 performance and confusion matrix for the prediction of UF, respectively. The best accuracy of 90 percent among the implemented pre-trained models was attained by the InceptionV3 transfer learning model. This model also performed equally better for the other performance metrics like F1 score, recall, and precision.

### 4.4. Proposed DPCNN

**Figure 13:**
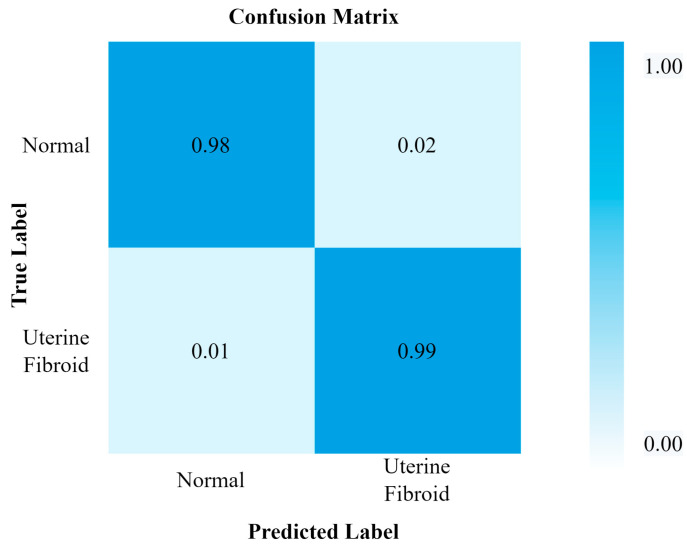
Confusion matrix for DPCNN.

**Figure 14:**
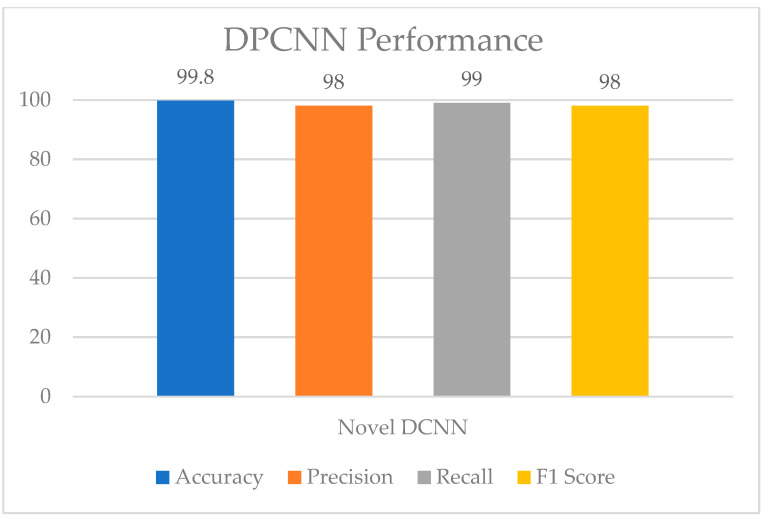
DPCNN performance.

Figure 13 and Figure 14 show the confusion matrix and performance of DPCNN to detect UF, respectively. The best-achieved accuracy of 99.8% was obtained by the proposed dual path DCNN.

One possible reason for achieving the remarkable performance could be attributed to the architecture of the proposed model, which used a dual pathway structure that allows for better feature extraction and representation. Additionally, data augmentation techniques such as random rotations, flips, and zooms were applied to the training set to increase its size and improve the generalization ability of the model. Furthermore, the proposed model was trained with a smaller learning rate and a higher number of epochs to prevent overfitting, which could lead to better generalization performance on the test data.

### 4.5. Comparison

**Figure 15:**
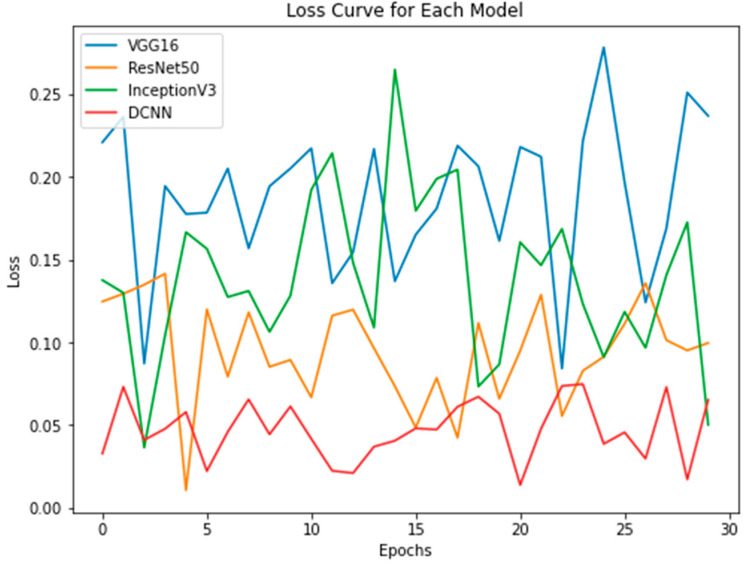
Loss curve of each model.

**Figure 16:**
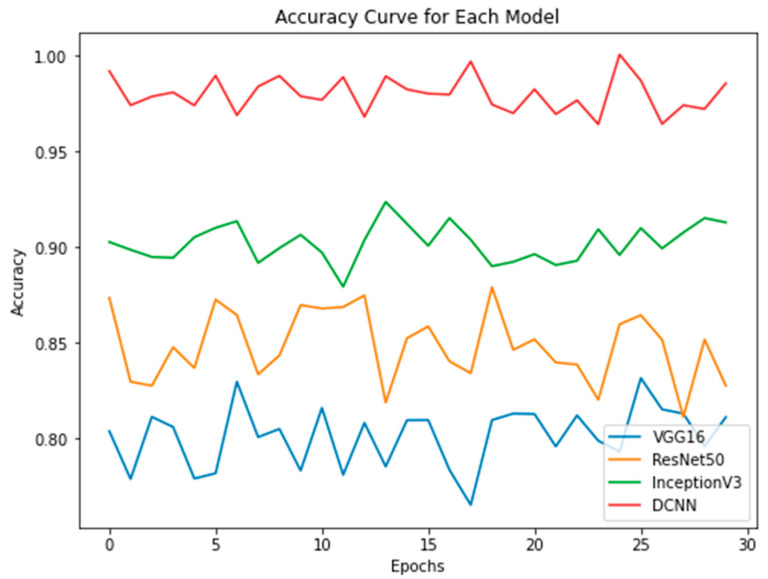
Accuracy of each model.

**Figure 17:**
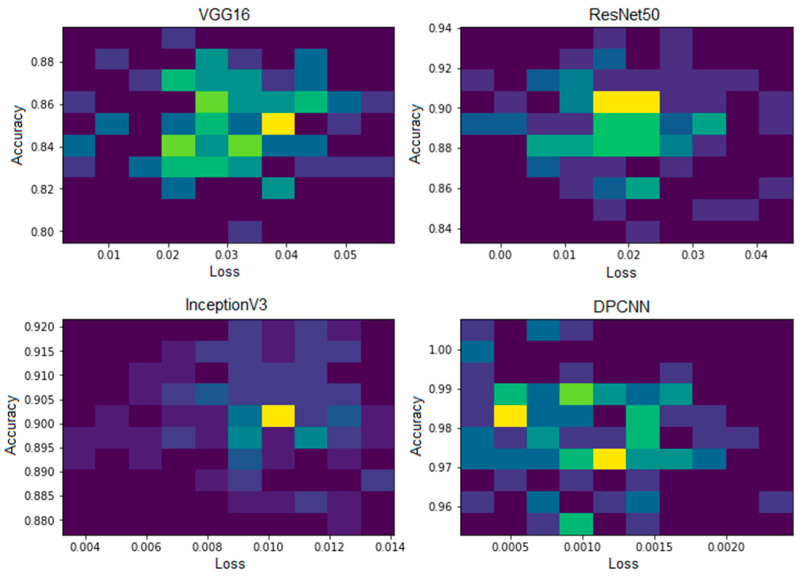
Performance comparison of each model (accuracy vs. loss).

The study evaluated the performance of four models, including novel DPCNN, InceptionV3, ResNet50, and VGG16, on the task of UF detection. Table 5 presents the accuracy achieved by each model, along with the optimizer used during training. The accuracy is reported as a percentage and represents the proportion of correctly classified images in the test dataset. Results showed that novel DPCNN outperformed the other models with an accuracy of 99.8%, while InceptionV3, ResNet50, and VGG16 achieved accuracies of 90%, 89%, and 85%, respectively. It is noteworthy that all models used the same dataset and preprocessing steps but varied in their architecture and optimizer. These results suggest that the proposed novel DPCNN model is highly effective for the automated detection of UF in ultrasound images. Additionally, Figure 15, Figure 16 and Figure 17 display the performance of each model during the training process. Figure 15 presents the loss curves of each model, where the *x*-axis represents the number of epochs, and the *y*-axis represents the loss value. The comparison of loss curves allows us to estimate the relative performance of each model in making predictions on the training data. Figure 16 displays the accuracy of each model during training, where the *x*-axis represents the number of epochs, and the *y*-axis represents the accuracy value. The comparison of the accuracy values of each model allowed us to evaluate their ability to make correct predictions on the training data. Figure 17 presents a performance comparison of each model in terms of accuracy versus loss, where each dot represents a single epoch of training for a particular model. The *x*-axis represents the loss value, and the *y*-axis represents the accuracy value. A model that achieves high accuracy and low loss is generally considered to be the best-performing model. These figures provide a visual representation of the performance of each model, allowing for a better understanding of the strengths and weaknesses of each approach.

**Figure 18:**
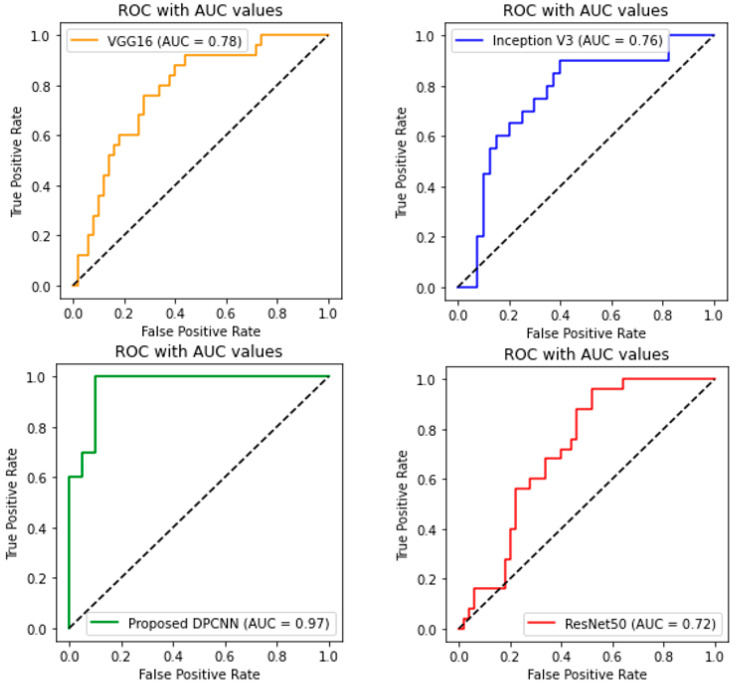
AUC score with ROC values for all implemented models.

A thorough evaluation based on AUC-ROC scores was conducted for each model. The proposed DPCNN architecture achieved the highest AUC-ROC score of 0.97. InceptionV3, ResNet50, and VGG16 attained an AUC score of 0.76, 0.72, and 0.78, respectively. Figure 18 provides a more comprehensive evaluation of our proposed approach.

## 5. Discussion

In this research, we compared the effectiveness of multiple cutting-edge DL architectures on the problem of detecting UF. A classification accuracy of 99.8 was attained by our proposed unique DPCNN architecture, which outperformed other state-of-the-art transfer learning models. Using data augmentation and transfer learning approaches, as well as the proposed model’s dual-pathway architecture, we showed that very accurate and reliable predictions can be made. Our method may help doctors in diagnosing UF tumors in earlier stages, which is one of its key benefits. It has the potential to boost diagnostic precision and efficiency, leading to quicker treatment and better health outcomes for patients. The proposed approach can be easily incorporated into current medical imaging systems, thereby increasing its use for healthcare professionals. The fields of medical imaging and computer-assisted diagnosis can also benefit from our research. Future studies on the use of ultrasound imaging for the detection of UF tumors can use our proposed model as a reference point. In addition, various medical imaging tasks, such as the detection of breast cancer tumors or lung nodules, can make use of the proposed model’s dual-pathway architecture. Our research has important social implications since it demonstrates the utility of artificial intelligence (AI) and DL to enhance healthcare delivery. Our proposed strategy has the potential to lessen the strain on healthcare systems while simultaneously increasing patient access to treatment. While AI has the potential to improve healthcare in many ways, it also brings up ethical and social concerns like data privacy and algorithmic partiality. To ensure that AI is used ethically in healthcare, future studies should concentrate on resolving these concerns. In conclusion, our research proves that ultrasound images may be used to detect UF tumors using this innovative deep convolutional neural network design. Our suggested model has the potential to aid clinicians in the early detection and diagnosis of UF tumors, hence increasing diagnostic precision and efficiency and enhancing the health of patients. Table 6 shows the results of a comparison between the suggested method and previous research.

The limitations of this research work are the relatively small size of the dataset used for training and testing the models. Although we have used data augmentation techniques to increase the dataset size, limited data can still affect the generalization of the proposed model to different populations. Therefore, future work can investigate the performance of the proposed model on larger and more diverse datasets to improve its robustness and generalization. Another limitation of our study is the use of only one type of imaging modality (ultrasound) for the detection of UF. Other imaging techniques such as magnetic resonance imaging (MRI) and computerized tomography (CT) can also be used for the detection of UF, and their integration with the proposed model can improve its diagnostic accuracy. However, due to the limited availability of annotated data for other imaging modalities, we focused only on ultrasound images in this study. Therefore, future work can explore other architectures and optimization techniques to further improve the model performance.

## 6. Conclusions

In conclusion, this research compared the performance of multiple deep convolutional neural network (DCNN) models for identifying uterine fibroid (UF) tumors in ultrasound scans. The experimental results demonstrated that the proposed innovative dual-path DCNN (DPCNN) model achieved a classification accuracy of 99.8% when trained with the stochastic gradient descend (SGD) optimizer, outperforming state-of-the-art architectures like VGG16, ResNet50, and InceptionV3. The results show that the proposed model’s dual-pathway architecture, when used in conjunction with data augmentation and transfer learning techniques, produced extremely accurate and trustworthy predictions. It is suggested that the proposed model’s efficacy be studied in more depth on a more comprehensive dataset in future research. The convergence rate and precision of the model can be enhanced by investigating other optimization strategies, such as Adam or AdaGrad. In addition, it would be helpful to create an interpretability framework to verify the clinical significance of the model’s learned features. Ultimately, the proposed model has the potential to aid doctors in the early detection and diagnosis of UF, which can increase diagnostic accuracy and efficiency. To provide a fair assessment of the study, its limitations related to the proposed method should be acknowledged. The use of a single dataset raises questions about the study’s capacity to generalize the model to different populations or imaging modalities. The study has not considered an essential factor in medical applications—the model interpretability.

## Figures and Tables

**Table 1:** Comparative studies with similar work.

References	Technique	Dataset	Accuracy
Dilna et al. [30]	Classification techniques	Ultrasound scanned uterus image	95.1%
Behboodi et al. [29]	UNet-based networks	US diagnostic imaging	86.2%
Li et al. [28]	Deep Learning	ChEMBL database	85%
Tang et al. [11]	AR-Unet Network	AR-Unet dataset	94.56%
Yang et al. [7]	Deep Neural Networks	Ultrasound images dataset	88.5%
Girija et al. [2]	Data Mining Techniques	450 patient datasets	89.54%
Huo et al. [5]	DL-based method	3870 ultrasound images dataset	87.45%

**Table 2:** The architecture of DPCNN.

1.	Input Layer: 224 × 224 RGB image First pathway: Convolutional Layer 1: 32 filters of size 3 × 3 with ReLU activation Output: 222 × 222 × 32
2.	Max Pooling Layer 1: Max pooling with size 2 × 2 Output: 111 × 111 × 32 Convolutional Layer 2: 64 filters of size 3 × 3 with ReLU activation Output: 109 × 109 × 64
3.	Max Pooling Layer 2: Max pooling with size 2 × 2 Output: 54 × 54 × 64
4.	Second pathway: Convolutional Layer 3: 32 filters of size 3 × 3 with ReLU activation Output: 222 × 222 × 32
5.	Max Pooling Layer 3: Max pooling with size 2 × 2 Output: 111 × 111 × 32
6.	Convolutional Layer 4: 64 filters of size 3 × 3 with ReLU activation Output: 109 × 109 × 64 Max Pooling Layer 4: Max pooling with size 2 × 2 Output: 54 × 54 × 64
7.	Merge pathways: Concatenation Layer: Merge the outputs of the two pathways Output: 54 × 54 × 128 Fully Connected Layers: Flatten Layer: Flatten the output of the previous layer Output: 373,248
8.	Dense Layer 1: 128 units with ReLU activation Output: 128 Dropout Layer: Randomly drop 50% of the units Output: 128 Dense Layer 2: 2 units with Softmax activation (for binary classification) Output: 2

**Table 3:** Layer-wise parameters description of proposed DPCNN.

Layer Type	Output Shape	Trainable Parameters	Non-Trainable Parameters
Input Layer (224 × 224 × 3)	224 × 224 × 3	0	0
Convolutional Layer 1 (32 filters, 3 × 3)	222 × 222 × 32	896	0
Max Pooling Layer 1 (2 × 2)	111 × 111 × 32	0	0
Convolutional Layer 2 (64 filters, 3 × 3)	109 × 109 × 64	18,496	0
Max Pooling Layer 2 (2 × 2)	54 × 54 × 64	0	0
Convolutional Layer 3 (32 filters, 3 × 3)	222 × 222 × 32	9248	0
Max Pooling Layer 3 (2 × 2)	111 × 111 × 32	0	0
Convolutional Layer 4 (64 filters, 3 × 3)	109 × 109 × 64	18,496	0
Max Pooling Layer 4 (2 × 2)	54 × 54 × 64	0	0
Concatenation Layer	54 × 54 × 128	0	0
Flatten Layer	373,248	0	0
Dense Layer 1 (128 units)	128	47,775,872	128
Dropout Layer (50% rate)	128	0	0
Dense Layer 2 (2 units)	2	258	2

**Table 4:** Performance assessment metrics and their formulas.

Metric	Formula
Accuracy	(TP+TN)/(TP+TN+FP+FN)
Precision	TP/(TP+FP)
Recall	TP/(TP+FN)
F1 score	$2\;*\;\frac{\left(precision\;*\;recall\right)}{\left(precision+recall\right)}$

where: TP = true positives; TN = true negatives; FP = false positives; FN = false negatives.

**Table 5:** Comparison of proposed DPCNN with different transfer learning-based models.

Model	Optimizer	Accuracy
Proposed DPCNN	SGD	99.8%
InceptionV3	SGD	90%
ResNet50	SGD	89%
VGG16	SGD	85%

**Table 6:** Comparison of proposed work with existing studies.

Reference	Dataset	Accuracy
[1]	Uterine Fibroids Images Data	88%
[3]	Uterine Fibroids Images Data	89%
[6]	Uterine Fibroids Images Data	94%
Proposed	Uterine Fibroids Images Data	99.8%

## Data Availability

Publicly available datasets were analyzed in this study. This data can be found here: https://www.kaggle.com/datasets/pavansanagapati/ultrasound-dataset (accessed on 1 February 2023).
